# Supplementary material for: Morality-Based Assertion and Homophily on Social Media: A Cultural Comparison Between English and Japanese Languages
Source: Front Psychol. 2021 Nov 5;12:768856. doi: 10.3389/fpsyg.2021.768856 (PMC8602074; doi:10.3389/fpsyg.2021.768856)
Supplement: Supplementary file 1 [file Data_Sheet_1.PDF]

Supplementary Material

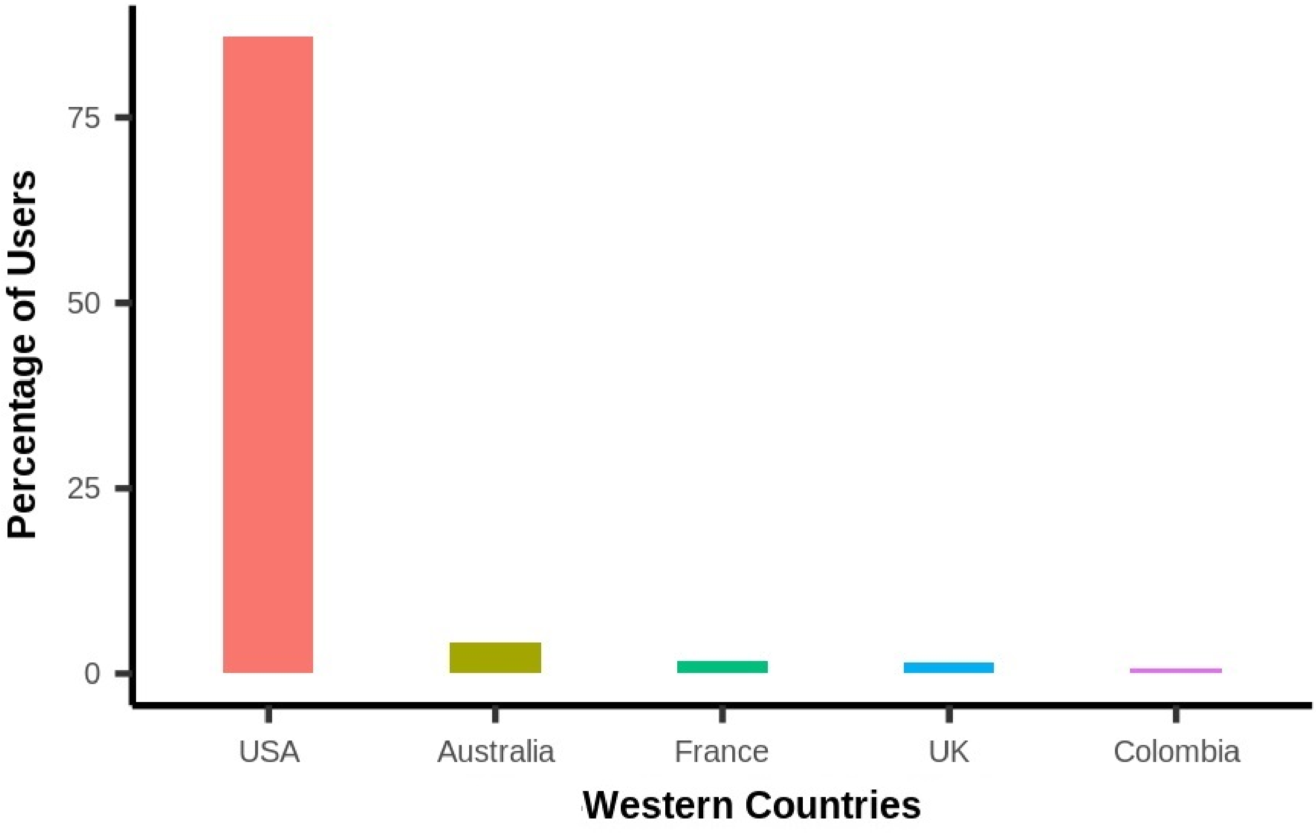

Figure S1. Top five countries based on the random selection of users (of English tweets) belonging to western countries.

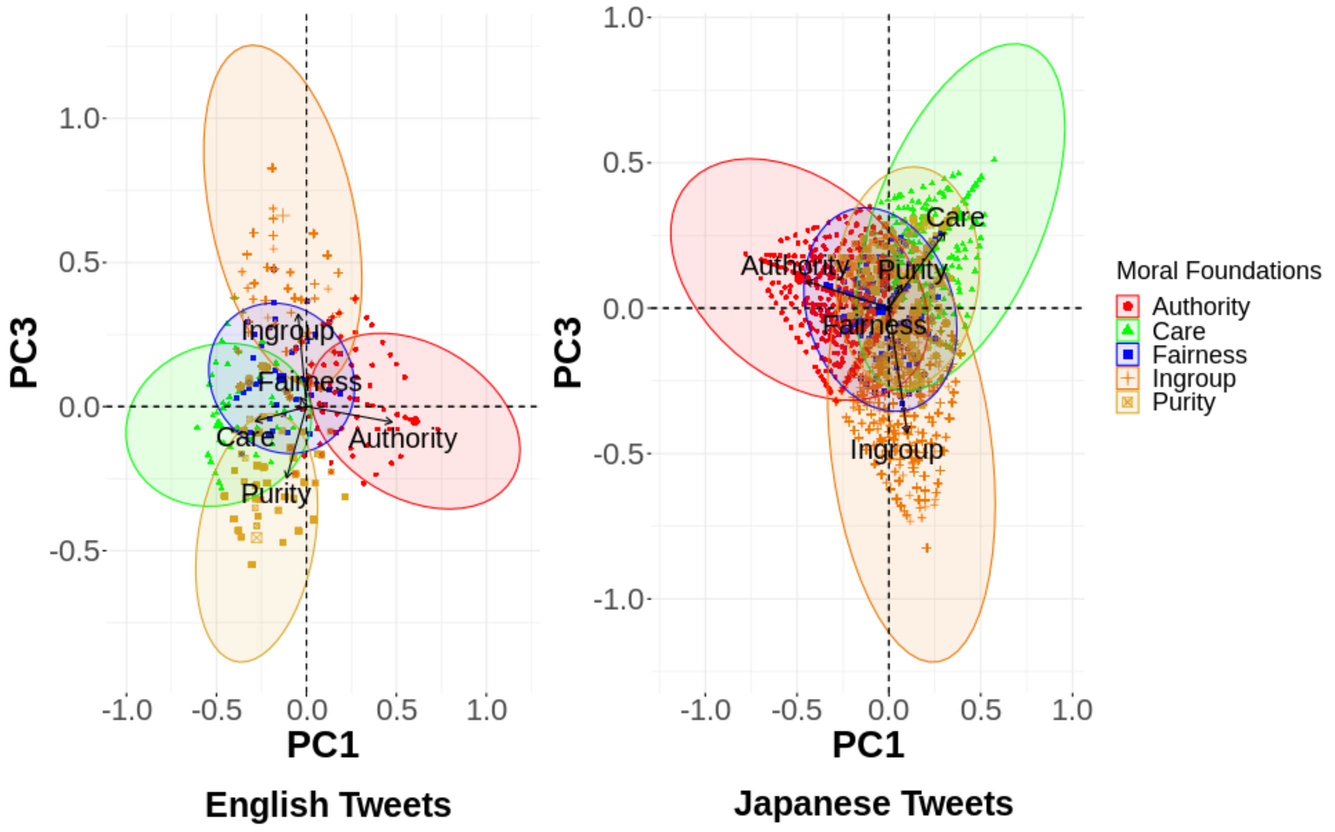

Figure S2. PCA Biplot with PC1 and PC3 for English and Japanese Moral Loadings.

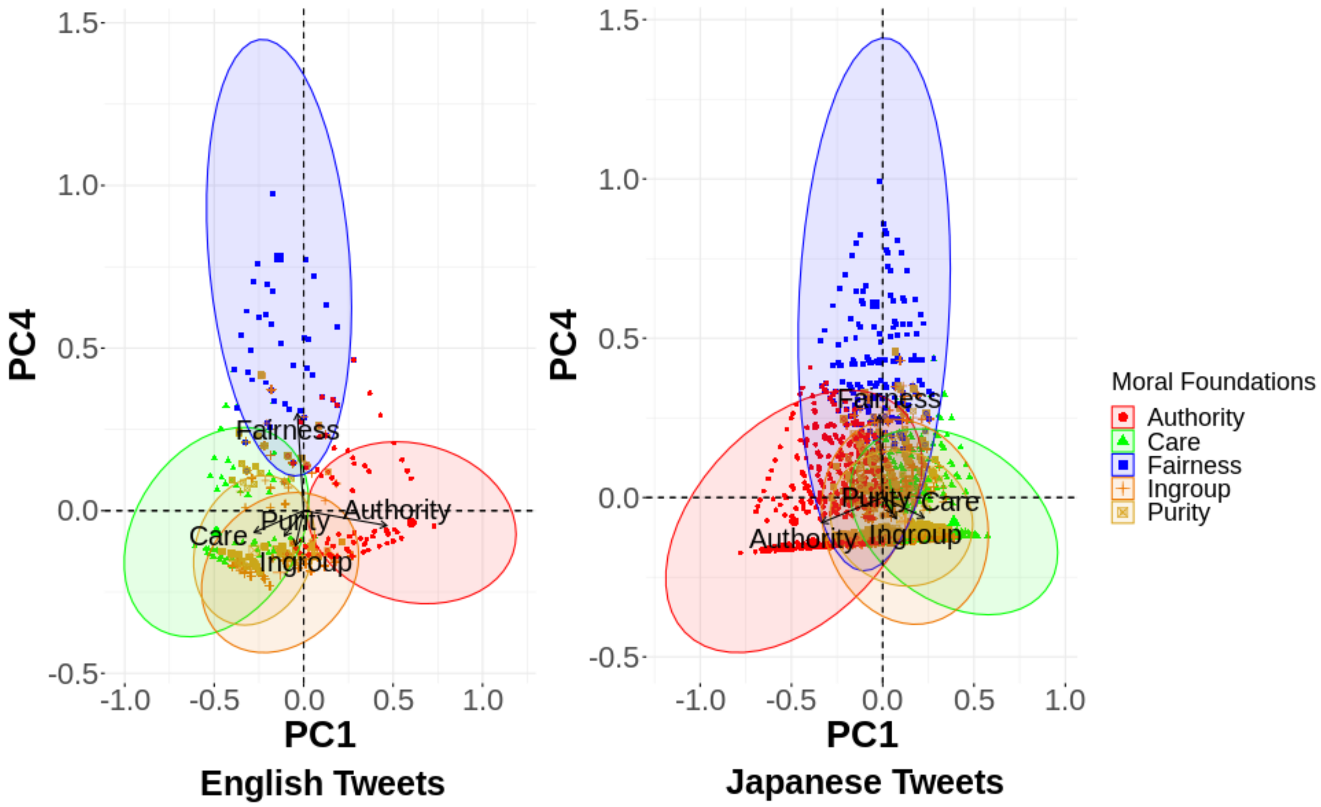

Figure S3. PCA Biplot with PC1 and PC4 for English and Japanese Moral Loadings.

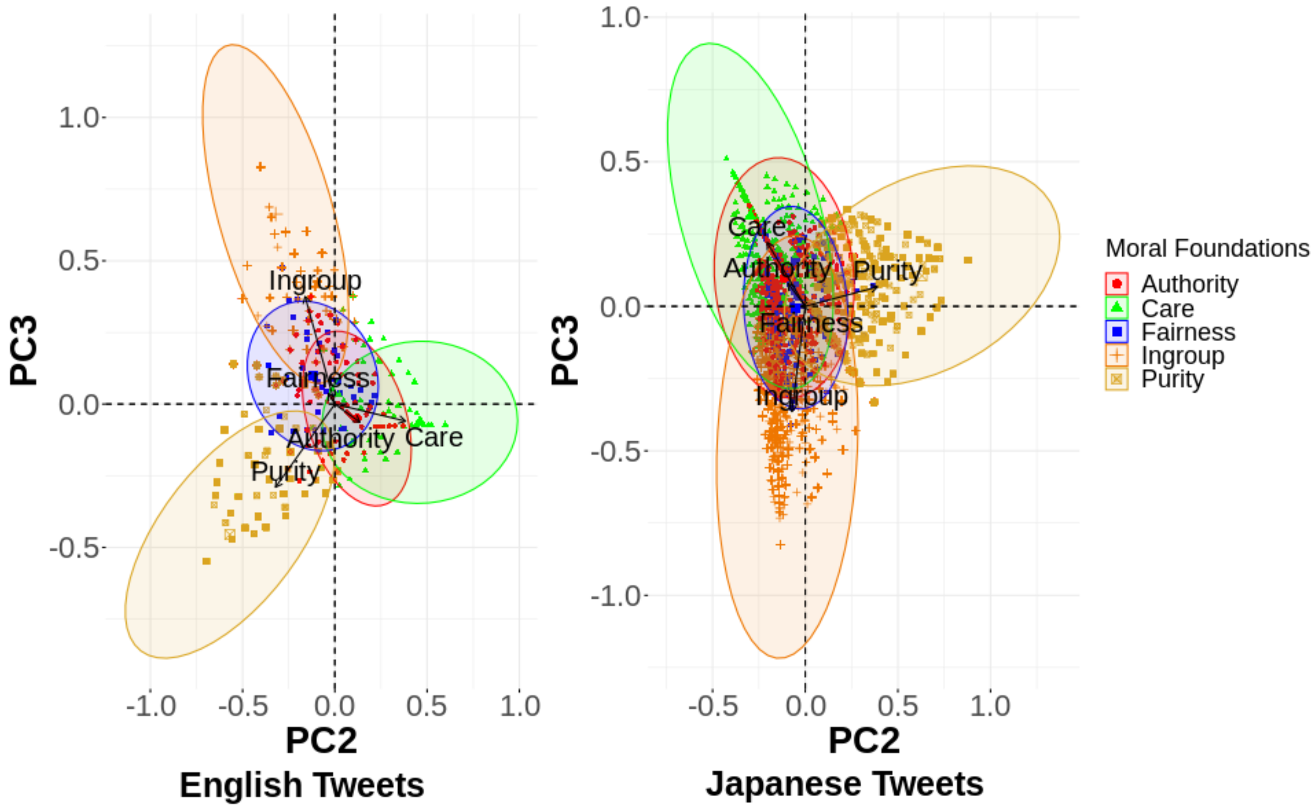

Figure S4. PCA Biplot with PC2 and PC3 for English and Japanese Moral Loadings.

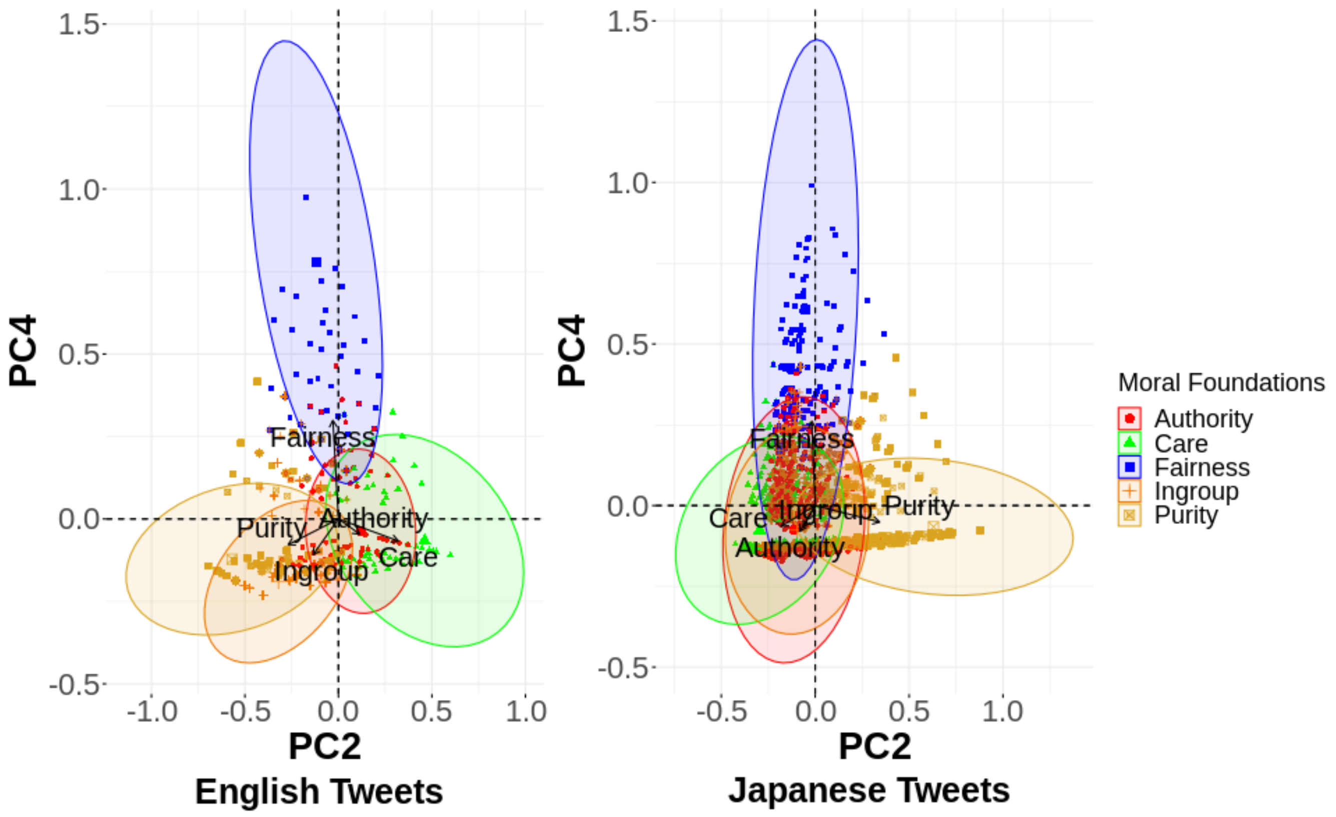

Figure S5. PCA Biplot with PC2 and PC4 for English and Japanese Moral Loadings.

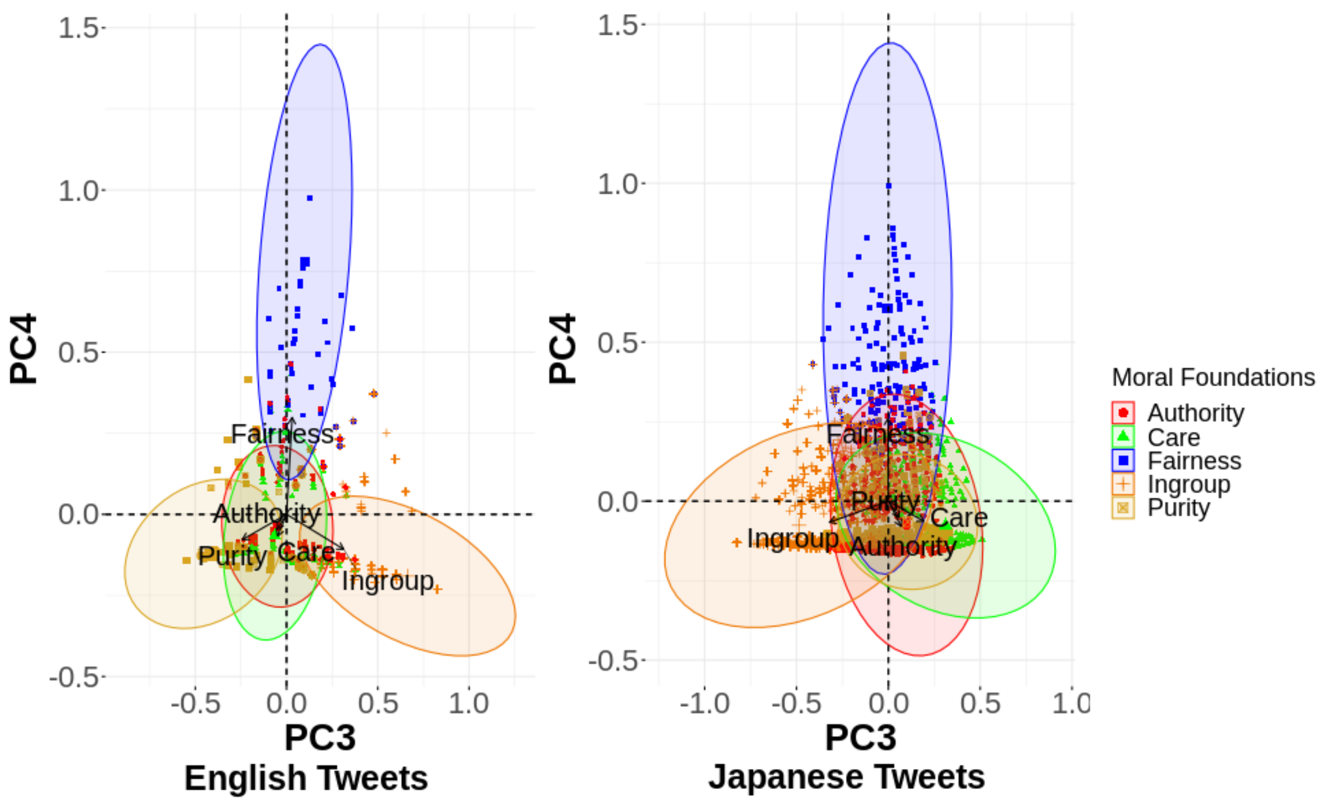

Figure S6. PCA Biplot with PC3 and PC4 for English and Japanese Moral Loadings.
